# Contextual effects of community capacity as a predictor for adolescent alcohol, tobacco, and illicit drug use: A multi-level analysis

**DOI:** 10.1016/j.ssmph.2023.101521

**Published:** 2023-09-25

**Authors:** Vera Birgel, Dominik Röding, Maren Reder, Renate Soellner, Ulla Walter

**Affiliations:** a Hannover Medical School, Institute for Epidemiology, Social Medicine and Health System Research, Carl-Neuberg-Str. 1, 30625, Hannover, Germany; b University of Hildesheim, Institute for Psychology, Universitätsplatz 1, 31141, Hildesheim, Germany

## Abstract

Adolescent substance use is a major public health issue that can result in enduring physical, psychological, and social consequences. This study seeks to examine the relationship between community capacity for prevention and the 4-week prevalence rate of substance use, including tobacco, alcohol, other drugs, and binge-drinking, among students in Germany ranging from grades 5 to 11. This study employed a cross-sectional design and used baseline data from 28 communities participating in the CTC-EFF study. The sample consisted of 7210 students who were surveyed about their substance use behavior. Additionally, 158 local key informants were surveyed on ten capacity domains, which included commitment, knowledge and skills, resources, leadership, inclusiveness, prevention collaboration, sectoral-collaboration, cohesion, problem-solving skills, and needs orientation. Furthermore, a total capacity score was calculated as the mean of the ten capacity domains. To examine the associations between community capacity and substance use behavior, logistic multi-level models were utilized. The analysis shows a negative association between community capacity (total score) and any substance use (OR = 0.28, 95% CI 0.12-0.56). Specifically, higher levels of total community capacity are associated with lower odds of alcohol use (OR = 0.30, 95% CI 0.13-0.80), tobacco use (OR = 0.09, 95% CI 0.01-0.60), and binge-drinking (OR = 0.67, 95% CI (0.46-0.99). Further analyses of distinct community capacity domains indicate that higher levels of sectoral-collaboration (OR = 0.62, 95% CI 0.37-0.97), knowledge and skills (OR = 0.74, 95% CI 0.40-0.79), resources (OR = 0.52, 95% CI 0.36-0.76), and problem-solving skills (OR = 0.71, 95% CI 0.36-0.89) are associated with lower odds of any substance use. The study findings suggest that community capacity is associated with substance use behavior, emphasizing the importance of capacity building in interventions targeting the reduction of substance use among adolescents.

## Highlights

• Total community capacity is associated with adolescent substance use patterns.
• Predominant negative associations across single capacity domains and substance use.
• First study in Germany connecting community capacity with youth substance use.

## 1 Introduction

Adolescent substance use, including alcohol, tobacco, and illicit drug use, is a critical public health concern worldwide with significant repercussions for physical and mental health, productivity, as well as economic well-being (Francis et al., 2015; WHO, 2021; Wilson, Battistich, Syme, & Boyce, 2002). Given the associations of adolescent substance use with adverse repercussions, such as compromised academic performance, heightened school dropout rates, and the emergence of behavioral issues, it is imperative to mitigate these detrimental effects to prevent ensuing health, social, and economic difficulties (Riva et al., 2018).

Building upon Bronfenbrenner's (1977) social ecological model (Bronfenbrenner, 1977), behavior is influenced not just by individual factors, but also by the wider societal context. While genetic predisposition, personal experiences, and peer influence are foundational to substance use (Griffin, Cleveland, Schlomer, Vandenbergh, & Feinberg, 2015), the impact of the community environment is pivotal (Das, Salam, Arshad, Finkelstein, & Bhutta, 2016). In particular, community interventions that focus on capacity building have demonstrated efficacy in preventing substance use (Chinman et al., 2005; Feinberg, Jones, Greenberg, Osgood, & Bontempo, 2010; Hawkins et al., 2009). Communities with high levels of capacity are more likely to implement and sustain effective prevention programs, provide access to resources that support healthy behaviors, and promote positive community norms regarding substance use (Chinman et al., 2005; Cole, 2022; Fagan, Hawkins, Farrington, & Catalano, 2018; Flewelling & Hanley, 2016; Oesterle et al., 2018).

Community capacity is a multidimensional concept, encompassing a community's ability to allocate resources, foster collaboration, and drive health-promoting initiative. Community capacity is shaped by several factors, including social and economic determinants (Chinman et al., 2005). Social determinants refer to the degree of social cohesion, civic engagement, and the presence of social networks that facilitate collaboration and resource sharing (Lee & Jung, 2018). Meanwhile, economic determinants represent the financial resources available and the degree of economic development enabling communities to invest in prevention efforts (Goodman et al., 1998).

However, despite a broad understanding of its key elements, community capacity remains a somewhat elusive concept, lacking a universally accepted definition (Simmons, Reynolds, & Swinburn, 2011). This study focuses on assessing the concept of community capacity for prevention (Goodman et al., 1998), which is conceptualized as one of the three main categories of community capacity, alongside generalized capacity (Chaskin, 2001), and organizational capacity (Hawe, Noort, King, & Jordens, 1997).

In a previously conducted scoping review, we identified nine dimensions of community capacity that have most commonly been utilized in prior studies to measure capacity in the field of prevention and health promotion (Birgel et al., 2023, Birgel et al., 2023). Our study involves a secondary data analysis of a Community Key Informant Interview (CKI) from the CTC-EFF-Study. By using the definitions of these domains, we were able to select items from the Community Key Informant Interview (CKI) that allow measurement of each dimension or partial aspects of the dimensions. The community capacity for prevention measures utilized for this study include: commitment, knowledge and skills, resources, leadership, needs orientation, prevention collaboration, sectoral-collaboration, cohesion, and problem-solving skills. Among these dimensions, the community's commitment in our study holds particular relevance to the concept of community readiness for prevention. Extensive research has highlighted the positive associations between community readiness and health outcomes (Gansefort, Brand, Princk, & Zeeb, 2018; Kostadinov, Daniel, Stanley, Gancia, & Cargo, 2015; Schröder, Schnabel, Hassel, & Babitsch, 2022). Community readiness, as introduced by the Tri-Ethnic Center for Prevention Research (Stith et al., 2006; Tri-Ethnic Center for Prevention Research, 2014), is closely related to community capacity as it reflects a community's preparedness and receptivity to address specific issues.

However, studies investigating the relationship between community capacity and youth substance use (Flewelling & Hanley, 2016) or other health outcomes (Jung & Viswanath, 2013; Lovell, Gray, & Boucher, 2017) are comparatively sparse, especially within the German context, as most of the existing studies have been conducted in the US. Only a few studies have examined the effects of community capacity on health outcomes in Germany, including a single case study analyzing capacity building within a health promotion intervention (Nickel, Süß, Lorentz, & Trojan, 2018), and a qualitative assessment of the interplay between capacity and physical activity (Loss et al., 2020).

Germany, with its comprehensive healthcare system, progressive prevention legislation, societal acceptance of low-alcohol consumption from age 16, well-structured communities and schools, and strong social welfare system, offers a distinct environment to explore the role of community capacity. In contrast to the US, where community volunteers frequently spearhead preventive efforts due to a historical emphasis on community mobilization to address societal concerns, Germany's focus on professionalized service delivery lessens this reliance. As a result, the impact of community capacity on youth substance use might vary between Germany and the US. While income disparities in the US often hinder community-led projects, especially in impoverished regions (Smeeding, 2005), Germany's solid welfare foundation, encompassing comprehensive social security, unemployment benefits, and universal healthcare (Eichhorst, 2015), might mitigate socio-economic barriers that impede community-led initiatives. Furthermore, Germany's prioritization of universal healthcare and expansive prevention legislation suggests a different role for community capacity (Busse & Blümel, 2014). Unlike in the US where community capacity might compensate for systemic gaps (Jonkman et al., 2009), it may act more as a complement to existing infrastructures in Germany.

This study explored the association between ten domains of community capacity and the prevalence of substance use, including tobacco, alcohol, illicit drug use, and binge drinking, among German students. Through a multilevel analysis that considers individual and community factors, this study aimed to gain a comprehensive understanding of the interplay between community capacity and adolescent substance use.

## 2 Methods

The present study used baseline data from the *Effectiveness of the community preventions system Communities That Care* (CTC-EFF) study, which was designed as a study to evaluate the effectiveness of the community-based prevention system Communities That Care (Hawkins & Catalano, 1992) in Germany (Röding et al., 2021).

### 2.1 Design and community recruitment

The design of the CTC-EFF study involved the recruitment of 42 small towns, rural communities, or districts of major cities across four German federal states (Bavaria, Lower Saxony, Rhineland-Palatinate, and Baden-Wuerttemberg); hereinafter referred to as communities. The assignment method was self-selection, where communities voluntarily chose to participate in the study. Sampling began in April 2020 with an invitation to all German communities that were in the early stages of implementing the Communities That Care (CTC) process. To be included in the trial, communities had to have at least one secondary school. The study employed an individual 1:1-matching procedure to identify comparable communities within the same federal state. Comparison communities were only eligible to participate in the study if (1) they had at least one secondary school, (2) they were not located in a county that implements CTC, and (3) they were not directly adjacent to a CTC-community. Overall, a sample of 21 matched pairs was recruited. For a detailed description of the sampling and data collection procedures employed in this study, please refer to Röding et al., 2021. Within this baseline data analysis, no evaluation based on intervention status was performed.

### 2.2 Sample and data collection

Of the initial 42 communities participating in the study, three communities opted to withdraw, and an additional 11 communities were excluded from the analysis as it was not possible to collect data from both students and key informants. Consequently, the final analysis included 28 communities, resulting in a sample size of 7210 students (fulfilling inclusion criteria) and 158 key informants. The data used for this analysis was collected between November 2021 and May 2022 (student-level data) and between May 2021 and November 2021 (community-level data) as part of the pre-intervention baseline assessment.

Adolescent substance use and socio-demographic details were gathered using the German adaptation of the CTC Youth Survey (Arthur, Glaser, & Hawkins, 2005). The construct validity of the German adaptation of CTC Youth Survey underwent a rigorous testing procedure in Lower Saxony-wide surveys (Soellner, Reder, & Frisch, 2018). The measures demonstrated high construct validity, including factor structure and concurrent validity (Reder, Runge, Schlüter, & Soellner, 2023). The survey was administered via an online questionnaire during a 45-min classroom period to students in grades 5, 6, 8, 10, and 11. Written informed consent was obtained from both parents and students. To mitigate the potential of social desirability bias and underreporting, an honesty question and a fictitious drug question were included in the Youth survey. Cases were excluded if a positive answer was given to the control question regarding the consumption of a fictitious drug (Phenoxydine, Pox, or PX), or if dishonesty was reported in most of the responses (Soellner et al., 2018). Specifically, exclusion was applied to 128 cases based on the honesty question, and 66 cases due to reported fictitious drug consumption.

To measure community capacity, a German context-adapted version of the Community Key Informant, obtained from the Community Youth Development Study (CYDS) (Hawkins et al., 2008), was utilized. The translated and abbreviated version underwent cognitive pretesting, multiple loops of revision, and initial validation analyses (Röding, Birgel, & Walter, 2023)

To generate a representative sample of community leaders, a two-stage process was employed. Firstly, the research team identified 538 individuals who held formal leadership positions (e.g., mayors, school principals, police officers, youth work leaders, or health officials) and invited them to participate in the survey. Subsequently, each surveyed key leader was requested to name two individuals in the community who were likely to have the most comprehensive knowledge of current prevention efforts. This snowball sampling technique led to the identification of a further 96 key informants that had not previously identified by the research team, bringing the total of eligible participants to 634. Of these 634 potential participants, 158 (25%) participated in the study. Of these 158 participants, 123 were identified during the first recruitment step, while 35 were recruited during the snowball-sampling. The reasons for non-participation varied among the remaining 476 community leaders, with 24.8% unable to be reached, 41.5% expressing a lack of interest in participating, and 33.7% feeling they lacked sufficient knowledge about the prevention work in their community. Participation varied from 3 to 10 leaders per community, with an average number of 5.6 key informants. The main method of data collection was computer-assisted telephone interviews, with an online survey option available.

## 3 Measures

### 3.1 Substance use

The study assessed the 4-week prevalence rate of alcohol, tobacco, and illicit drug use, as well as binge drinking in grades 5 to 11. Students were asked how many times they had used alcohol, tobacco, or illicit drugs (i.e., marijuana, psychedelics, MDMA, stimulants, and cocaine) during the past 4 weeks (e.g., “On how many occasions (if any) have you had beer, wine, or hard liquor during the past 4 weeks?“). Responses were dichotomized as 0 (no use, including lifetime abstainers) or 1 (any use). Binge drinking was defined as the consumption of 5 or more drinks on a single occasion. The responses regarding binge drinking were dichotomized as 0 (no occurrence) or 1 (at least one episode).

### 3.2 Community capacity

The study used ten domains to assess community capacity for prevention and health promotion, identified through a scoping review (Birgel, Decker, et al., 2023). Based on the definitions of these domains, items from the Community Key Informant (CKI) were identified that allowed measurement of each domain or dimensions of the domain, notwithstanding that the CKI was not originally designed for comprehensive community capacity assessment. All items were rated on a 4-point Likert scale, with higher values indicating a higher capacity. The ten capacity domains were then calculated as the mean of their underlying items. Beforehand, all items were coded so that higher values indicate higher capacity. Given that community capacity is a collective attribute, the ratings of the key informants within each community were averaged to obtain a mean value for each community. The ten domains were:

1. Prevention Collaboration (9 items): Measures the quality of prevention collaboration (9 items) (e.g., *In your community, each organization has a clear role in implementing the local prevention plan*).
2. Sectoral Collaboration (10 items): Assesses the degree of interagency collaboration across ten community sectors (i.e., *secondary schools, youth work, youth centers, social work, drug counseling centers, police, child protection, youth recreation, juvenile justice, and human service agencies*).
3. Commitment (2 items): Measures whether community members are committed to addressing community issues and have the ability to influence drug, alcohol, and tobacco abuse in the community.
4. Knowledge & skills (4 items): Evaluates the knowledge and skills of community members to accomplish positive community development (e.g., *Generally, people in my community are knowledgeable about local prevention efforts*).
5. Resources (4 items): Examines deficiencies in several resource areas, including lack of financial resources, lack of human resources, and lack of support in the community.
6. Leadership (1 item): Determines to what extent a lack of leadership has been a barrier to prevention-related activities in the community.
7. Needs orientation (1 item): Assesses whether the community selects new prevention activities based on its needs.
8. Cohesion (1 item): Evaluates whether community members feel connected and share a sense of identity.
9. Problem-solving skills (4 items): Assesses the ability and capacity of the community to work together to identify and solve problems (e.g*., In the last year, my community has been successful at addressing social problems*).
10. Inclusiveness (1 item): Examines the proportion of individuals from diverse ethnic and cultural backgrounds who participate in prevention planning and implementation.

Additionally, a total capacity score was calculated as the mean of all underlying capacity domains. For more details on the capacity constructs and related items, please refer to Supplementary Table 1.

### 3.3 Student and community characteristics

Student characteristics used as covariates in analyses included grade, gender, and school type. School type was dichotomized into secondary school preparing for university attendance (“Gymnasium”) vs. “other school type” due to the diverse German school system across federal states. “Gymnasium” is generally considered to be academically rigorous and emphasizes a comprehensive education. The category “other” encompasses various school types, namely Hauptschule (basic general education), Realschule (broader academic focus), and Gesamtschule (integration of Hauptschule and Realschule elements).

Variables measuring community demographic characteristics were obtained from 2019 data provided by the Federal Institute for Research on Building, Urban Affairs, Spatial Development (BBSR) and consisted of (a) the total population of the community and (b) the community type (central vs. peripheral) (BBSR Bonn, 2019). Central communities are defined as high-density cities with strong economies and good infrastructure, while peripheral communities are low-density areas with weaker economies and fewer development opportunities, located in rural regions or on the outskirts of urban areas (BBSR Bonn, 2019).

### 3.4 Missing data

Of the 7210 students who met inclusion criteria, 652 (9.0%) had missing data on all substance use items. Consequently, the final analysis sample comprised 6558 students. No significant differences were found on gender, grade, or school type (p > .05) between the 6558 students included in the analysis and the 652 students with missing data.

With respect to the capacity constructs, the item non-response rate ranged from 0% to 9.8%. Key leaders with missing data did not differ from those without missing data in age, gender, education, or length of residency in the community (p >.05). To handle missing values for the community-level constructs, the community-specific mean for each item was used.

### 3.5 Statistical analysis

Prior to the present analyses, the validity and reliability of the community capacity scales was evaluated, as described in Birgel, Walter, & Röding, 2023. Structural validity of the ten capacity constructs was investigated through an exploratory factor analysis (EFA) and confirmatory factor analysis (CFA). The EFA was conducted as principal component analyses using the varimax method. An EFA considering all capacity items identified 10 factors with an eigenvalue greater than 1. These factors represent the ten capacity domains with low imprecision. The KMO criterion for this model is 0.718. Commonality is above 0.5 for 33 of these 37 indicators and between 0.4 and 0.5 for the remaining four indicators (see Supplementary Table 2). The CFA model provided a good fit, χ2 (73, n = 158) = 275.70, p = .138, CFI = 0.96, TLI = 0.96, RMSEA = 0.03 (see Supplementary Table 3). The internal consistency of the community capacity scales and subscales was good to acceptable, with Cronbach's alpha ranging from 0.69 to 0.87 (see Supplementary Table 1). These results are comparable to those of other studies that surveyed key informants on community level characteristics (Arthur et al., 2005; Lovell et al., 2017; Shapiro, Oesterle, & Hawkins, 2015).

Multi-level binary logistic regression models with random intercept were used to analyze the effect of community capacity on the 4-week prevalence of any substance, alcohol and tobacco use (0 = no use, 1 = any use), as well as binge drinking (0 = no occurrence, 1 = at least one time). The random intercept accounted for clustering of students (Level 1) within communities (Level 2). For each substance use outcome, eleven models were calculated. The first model included an aggregate capacity score, which was calculated as the mean of all capacity domains. Subsequently, each of the ten capacity domains was individually included in separate models. Due to observed multicollinearity among capacity domains (with correlations exceeding 0.7 for some pairs as noted in Supplementary Table 4), and limited community-level sample size, a simultaneous inclusion of all capacity domains was not feasible. In response, for each model that included a single capacity domain, a composite measure based on the remaining nine domains was controlled for. This approach allowed us to mitigate issues related to overfitting and multicollinearity (Gelman & Hill, 2012; Hox, 2010; Maas & Hox, 2004, 2005).

## 4 Results

### 4.1 Descriptive statistics

#### 4.1.1 Individual-level characteristics

Out of the 6558 students surveyed, 48.2% identified as male, 50.6% as female, and 1.2% as non-binary (Table 1). Regarding grade distribution, 24.7% of the students were in grade 8, 24.3% in grade 6, and 21.9% in grade 10. Among the surveyed students, slightly over half (52.0%) attended a “Gymnasium".

**Table 1:** Descriptive statistics.

Characteristics		Range			
	*n*	Min	Max	Percent	*M (SD)*
*Individual level (level-1, N = 6558)*					
Gender					
Male	3164			48.2	
Female	3318			50.6	
Nonbinary	76			1.2	
Grade					
5	1293			19.7	
6	1594			24.3	
8	1617			24.7	
10	1434			21.9	
11	620			9.5	
School type					
“Gymnasium”	3409			52.0	
Other school type	2503			48.0	
Any substance use past 4-weeks					
No usage	3916			59.7	
Usage	2642			40.3	
Alcohol use past 4-weeks					
No usage	4141			61.68	
Usage	2503			38.2	
Binge drinking past 4-weeks					
No occurrence	4953			77.1	
At least one occasion	1469			22.9	
Tobacco use past 4-weeks					
No usage	5547			85.8	
Usage	929			14.2	
Illicit drug use past 4-weeks					
No usage	6183			96.9	
Usage	203			3.1	
*Community level (level-2, J = 28)*					
Population size		3629	30,689		15,407 (6091)
Community type					
Central	18			64,3	
Peripheral	10			35,7	
Community capacity (total score)^a^^,^^b^		1.00	4.00		2.36 (.14)
Prevention Collaboration^b^		1.00	4.00		2.58 (.38)
Sectoral-Collaboration^b^		1.00	4.00		2.48 (.28)
Knowledge^b^		1.00	4.00		1.91 (.20)
Resources^b^		1.00	4.00		2.74 (.38)
Leadership^b^		1.00	4.00		2.62 (.31)
Cohesion^b^		1.00	4.00		1.62 (.27)
Problem-solving skills^b^		1.00	4.00		1.93 (.19)
Inclusiveness^b^		1.00	4.00		2.28 (.29)
Needs orientation^b^		1.00	4.00		3.27 (.36)
Commitment^b^		1.00	4.00		2.12 (.51)

Note: *M* = Mean, *SD* = standard deviation.

a The total community capacity score was calculated as the mean of all capacity domains.

b Aggregate variables.

#### 4.1.2 Community-level characteristics

The analysis included 28 communities, ranging from 3629 to 30,689 inhabitants (M = 15,407, SD = 6091). Most communities were central (64.3%) rather than peripheral (35.7%). Community capacity was assessed using a 4-point Likert scale, with higher values indicating greater capacity. The overall capacity score, calculated as the mean of all capacity items, was found to be 2.33 (SD = 0.14). This score falls in the middle of the potential range, indicating a generally moderate level of community capacity across the communities.

### 4.2 Associations between community capacity and the prevalence of substance use

The results of the multi-level logistic regression analysis revealed a significant negative association between community capacity, as measured by the total score, and any substance use among students (OR = 0.28, 95% CI 0.12-0.56). This finding suggests that a one-unit increase in the total capacity score is associated with a 72% decrease in the odds of any substance use among students. Specifically, higher levels of total community capacity were significantly associated with reduced odds of alcohol (OR = 0.30, 95% CI 0.13-0.80) and tobacco use (OR = 0.09, 95% CI 0.01-0.60) (Table 2). However, there was no statistically significant association between total community capacity and illicit drug use (OR = 0.60, 95% CI 0.21-1.75). This suggests that while there may be a trend towards a protective effect of community capacity, this effect is not statistically significant, and the estimate's confidence interval is wide, indicating substantial uncertainty in this association. Likewise, the odds ratio for the association between total community capacity and binge drinking was 1.07, with a wide 95% confidence interval (0.32 - 3.52, p = .914), indicating a non-statistically significant association and a considerable uncertainty around the estimate.

**Table 2:** Association between total community capacity and substance use in students showing odds ratios for any substance use.

	any substance use OR (95% CI)	any alcohol use OR (95% CI)	any tobacco use OR (95% CI)	any illicit drugs use OR (95% CI)	any binge drinking OR (95% CI)
Constant	0.17 (0.07-0.43) p = .036	0.12 (0.04-0.31) p = .043	0.14 (0.05-0.37) p = .039	0.20 (0.08-0.51) p = .005	0.20 (0.09-0.45) p = .011
*Community characteristics*					
Community capacity (total score)^a^	0.28 (0.12-0.6) p = .008	0.30 (0.13-0.80) p = .009	0.09 (0.01-0.60) p = .010	0.60 (0.21-1.75) p = .351	1.07 (0.32-3.52) p = .914
Population size	1.00 (1.00-1.00) p = .634	1.00 (1.00-1.00) p = .915	1.00 (1.00-1.00) p = .192	1.00 (1.00-1.00) p = .272	1.00 (1.00-1.00) p = .295
Community type “central”	0.77 (0.57-1.05) p = .148	0.77 (0.57-1.05) p = .100	0.77 (0.54-1.11) p = .147	1.12 (0.90-1.41) p = .326	0.67 (0.46-0.99) p = .044
*Student characteristics*					
Male^b^	1.22 (1.08-1.37) p = .002	1.19 (1.06-1.35) p = .005	1.13 (0.97-1.32) p = .126	1.20 (0.95-1.50) p = 117	1.21 (1.05-1.39) p = .008
Grade (ref. 11)					
5	0.03 (0.02-0.03) p>.001	0.03 (0.02-0.03) p>.001	0.03 (0.02-0.04) p>.001	0.26 (0.17-0.39) p>.001	0.02 (0.01-0.02) p>.001
6	0.04 (0.03-0.04) p>.001	0.03 (0.03-0.04) p>.001	0.05 (0.02-0.06) p>.001	0.26 (0.25-0.52) p>.001	0.02 (0.02-0.04) p>.001
7	0.14 (0.10-0.16) p>.001	0.13 (0.10-0.16) p>.001	0.23 (0.07-0.19) p>.001	0.33 (0.52-1.19) p>.001	0.12 (0.08-0.24) p>.001
8	0.58 (0.44-0.72) p>.001	0.56 (0.44-0.72) p>.001	0.71 (0.28-0.80) p = .003	0.72 (0.63-1.51) p = .056	0.50 (0.44-1.14) p>.001
School type “Gymnasium”	0.87 (0.75-1.01) p = .068	1.04 (0.90-1.20) p = .607	0.37 (0.30-0.44) p>.001	0.64 (0.80-1.02) p = .066	0.54 (0.44-0.76) p>.001
*n*	6470	6476	6421	6482	6346

*Note*: OR = odds ratio; CI = confidence interval.

a The total capacity score is calculated as the mean of all capacity items.

b The category “non-binary” was not included in the multivariate analyses due to the small group size (n = 76).

Further analysis of the distinct capacity domains revealed significant associations between several domains and any substance use (Table 3). Specifically, higher levels of sectoral collaboration (OR = 0.62, 95% CI 0.37-0.97), knowledge and skills (OR = 0.74, 95% CI 0.40-0.79), resources (OR = 0.52, 95% CI 0.36-0.76), problem-solving skills (OR = 0.71, 95% CI 0.36-0.89), and commitment (OR = 0.75, 95% CI 0.50-0.98) were all linked to lower odds of any substance use. For alcohol use, significant associations were found with prevention collaboration (OR = 0.66, 95% CI 0.37-0.98), sectoral collaboration (OR = 0.69, 95% CI 0.43-0.79), commitment (OR = 0.81, 95% CI 0.58-0.98), and resources (OR = 0.65, 95% CI 0.46-0.93). Regarding tobacco use, higher sectoral collaboration (OR = 0.63, 95% CI 0.35-0.93), knowledge (OR = 0.67, 95% CI 0.37-0.98), resources (OR = 0.53, 95% CI 0.34-0.82), problem-solving skills (OR = 0.51, 95% CI 0.25-0.95), and commitment (OR = 0.76, 95% CI 0.31-0.87) were linked to reduced odds. Illicit drug use was associated with prevention collaboration (OR = 0.55, 95% CI 0.16-0.96), sectoral collaboration (OR = 0.68, 95% CI 0.44-0.89), resources (OR = 0.53, 95% CI 0.34-0.82), problem-solving skills (OR = 0.51, 95% CI 0.25-0.89), and commitment (OR = 0.76, 95% CI 0.51-0.96). Binge drinking was only associated with resources (OR = 0.69, 95% CI 0.44-0.95). No significant associations were found between the domains of needs orientation, cohesion, and leadership with any of the substance use outcomes.

**Table 3:** Association between single community capacity domains and substance use in students showing odds ratios for any use.

	any substance use OR (95% CI)	any alcohol use OR (95% CI)	any tobacco use OR (95% CI)	any illicit drugs use OR (95% CI)	any binge drinking OR (95% CI)
Community capacity domains^a^					
Prevention collaboration	0.81 (0.44-1.49) p = .348	0.66 (0.37-0.98) p = .043	1.08 (0.56-2.07) p = .815	0.55 (0.16-0.96) p = .038	0.77 (0.38-1.57) p = .470
Sectoral collaboration	0.62 (0.37-0.97) p = .035	0.69 (0.43-0.79) p = .048	0.63 (0.35-0.93) p = .042	0.68 (0.44-0.89) p = .048	0.85 (0.46-1.58) p = .611
Knowledge	0.74 (0.40-0.79) p = .023	0.92 (0.50-1.71) p = .797	0.67 (0.37-0.98) p = .048	0.76 (0.34-1.59) p = .468	1.11 (0.53-2.34) p = .774
Resources	0.52 (0.36-0.76) p<.001	0.65 (0.46-0.93) p = .018	0.53 (0.34-0.82) p = .005	0.53 (0.34-0.82) p = .005	0.69 (0.44-0.95) p = .008
Leadership	1.02 (0.55-1.95) p = .955	0.70 (0.38-1.29) p = .258	1.05 (0.61-1.46) p = .573	1.22 (0.61-2.01) p = .573	0.91 (0.42-2.42) p = .805
Cohesion	0.93 (0.61-1.40) p = .714	1.00 (0.67-1.51) p = .967	0.89 (0.57-1.40) p = .612	0.89 (0.57-1.40) p = .612	1.05 (0.66-1.66) p = .830
Problem-solving skills	0.71 (0.36-0.89) p = .039	0.87 (0.48-1.57) p = .637	0.51 (0.25-0.95) p = .048	0.51 (0.25-0.89) p = .48	0.96 (0.47-1.98) p = .915
Inclusiveness	0.66 (0.31-1.40) p = .427	0.66 (0.30-1.25) p = .724	0.97 (0.47-2.01) p = .932	0.86 (0.58-1.34) p = .564	0.67 (0.32-1.40) p = .283
Needs orientation	1.07 (0.67-1.72) p = .291	1.04 (0.66-1.62) p = .874	1.15 (0.66-1.95) p = .627	1.15 (0.66-1.95) p = .627	1.74 (0.92-3.28) p = .090
Commitment	0.75 (0.50-0.98) p = .050	0.81 (0.58-0.98) p = .049	0.76 (0.31-0.87) p = .036	0.76 (0.51-0.96) p = .036	1.18 (0.79-1.76) p = .420
*n*	6470	6476	6421	6482	6346

*Note*: OR = odds ratio; CI = confidence interval.

a Models adjusted for a composite measure based on the remaining capacity domains, gender, grade, school type, population size, and community type.

The Level-1 control variables, including gender, grade level, and school type, were significantly associated with substance use. Male students had higher odds of substance use (OR = 1.22, 95% CI 1.08-1.37), alcohol consumption (OR = 1.19, 95% CI 1.06-1.35), and binge drinking (OR = 1.21, 95% CI 1.05-1.39) than female students. Additionally, higher grade levels correlated with increased odds of substance use across all categories (Table 2). Students of the “Gymnasium”, representing a higher level of education, had lower odds of tobacco use (OR = 0.37, 95% CI 0.30-0.44) and binge drinking (OR = 0.54, 95% CI 0.44-0.76).

The level-2 control variable, population size, was not found to be significantly associated with any of the substance use outcomes. However, an association was found between community type and binge drinking. Specifically, students residing in a central community exhibited lower odds of engaging in binge drinking compared to students residing in a peripheral community (OR = 0.67, 95% CI 0.46-0.99).

## 5 Discussion

Adolescent substance use is a critical public health issue. An array of research has considered the influence of familial, social, and individual factors on the risk of adolescent substance use (Whitesell et al., 2013). While research, including the Communities That Care (CTC) system, has explored community-level factors, the role of community capacity remains largely unexplored. Community capacity, as an indicator of a community's ability to mobilize resources and tackle public health issues, can significantly impact the success of prevention initiatives (Foster-Fishman et al., 2006; Kegler, Rigler, & Honeycutt, 2010). The present study aimed to investigate the association between community capacity and adolescent substance use.

This study found associations between community capacity and substance use outcomes. The composite measure of community capacity exhibited significant associations with any substance use, as well as alcohol and tobacco use. Despite these observed trends, regarding the associations with illicit drug use and binge drinking, our findings were not statistically significant. In the case of illicit drug use, a negative, non-significant relationship was found between higher community capacity levels and the likelihood of engaging in such behavior. However, it is essential to consider that only a small proportion of the respondents reported illegal drug use, which may have introduced uncertainty to the results. The wide confidence interval further emphasizes the need for caution in interpreting this finding. Similarly, for binge drinking, the odds ratio was close to 1 and had a wide confidence interval, indicating a lack of statistically significant association and substantial uncertainty.

On examining the distinct capacity measures, we found various domains of community capacity to be significantly associated with different categories of substance use. Specifically, prevention collaboration, sectoral collaboration, knowledge and skills, resources, problem-solving skills, and commitment exhibited significant associations with different substance use outcomes. In our study, prevention collaboration, sectoral collaboration, and resources showed associations with multiple substance use outcomes. These factors align with the World Health Organization's Setting Approach, which recognizes the crucial role of communities in promoting public health (WHO, 1986). Furthermore, the study found that knowledge and skills, problem-solving skills, as well as commitment are significantly associated with adolescent substance use.

However, our results showed a unique pattern for binge drinking, with only the 'resources' domain associated with its decreased risk. This deviation might indicate that binge drinking among adolescents, often driven by peer influence and social norms, has different dynamics than other substance use behaviors (Borsari & Carey, 2001).

Despite some associations not reaching statistical significance, our analysis identified that 31 out of the 40 associations between community capacity domains and the 4-week prevalence of substance use (including any substance use, alcohol use, tobacco use, and illicit drug use) were negative. Similarly, 6 out of the 10 associations with binge drinking were negative. This overall pattern of predominantly negative associations suggests a modest yet overarching association between community capacity and adolescent substance use. However, no significant associations were found between needs orientation, leadership, inclusiveness, or cohesion with any of the substance use outcomes. We recognize that inclusiveness, needs orientation, and leadership were measured using only one item, which limited the comprehensive assessment of these aspects. For instance, the quality of leadership within a community may play a critical role in community capacity. Hence, further research is necessary to assess these domains more comprehensively and gain a better understanding of their role in the context of substance use prevention.

Our study aligns with prior research that has emphasized the role of community capacity in promoting positive health outcomes (Nagorcka-Smith et al., 2022), such as improved self-rated health (Jung & Viswanath, 2013) and reduced alcohol use (Flewelling & Hanley, 2016). Both in the German context and as noted in Flewelling & Hanley's work on the U.S., collaborative efforts seem to be significant. Our findings indicate that domains such as prevention collaboration and sectoral collaboration were notably associated with most substance use outcomes, emphasizing the importance of prevention-focused partnerships bridging various sectors within the community. This perspective is mirrored in Flewelling and Hanley's (2016) research, which pointed to the value of internal organization, structural considerations, and community connections in relation to adolescent alcohol consumption.

However, a distinct difference emerged when considering the role of resources. In the German setting, resources were observed to have a strong association with overall substance use and maintained this association across all individual substance use outcomes. This starkly contrasts the U.S. perspective presented by Flewelling & Hanley, where funding showed only a marginal association with a single substance use outcome. This pronounced emphasis on resources in our findings suggests a potential orientation in Germany towards structured, professionalized health interventions. Conversely, the U.S., with its historical leaning towards community-driven initiatives, appears to be more dependent on grassroots, volunteer-centric efforts in health promotion. In Germany, community capacity, and particularly resources, seem to play a complementary role, potentially enhancing established professional mechanisms. Meanwhile, in the U.S., these capacities might be more oriented towards addressing and bridging systemic gaps.

Another finding of the study is that there was no significant association between community size or type with adolescent substance use, except for binge drinking. This result is consistent with previous research indicating that community-level factors, such as social norms and resource availability, may be more important than community size or type in shaping adolescent substance use behavior (Chuang, Ennett, Bauman, & Foshee, 2005; Wagenaar, Tobler, & Komro, 2010). This is an encouraging finding, as it suggests that public health interventions focused on capacity building might have a significant impact on reducing adolescent substance use, regardless of firmer community structures.

While the study provides insights into associations between community capacity and adolescent substance use, there are limitations that need to be considered when interpreting the results. Firstly, being cross-sectional, the study only captures data at one time-point. Therefore, it is impossible to determine whether heightened levels of community capacity result in a reduction in substance use or whether the opposite is true. Moreover, the relationship noted between community capacity and substance use may not reflect a causal pathway, but rather an underlying network of unmeasured factors influencing both. Moving forward, we are in the process of initiating the second wave of the CTC-EFF study and planning additional future waves. This longitudinal approach will enhance our capacity to track changes over time and thereby more robustly interrogate the relationship between community capacity and substance use. This strategy has the potential to mitigate the inherent limitations of cross-sectional analysis by allowing us to more definitively ascertain the causality and directionality of the associations observed in our initial investigation. Secondly, it is important to acknowledge that the study relied on self-report data, which may be subject to social desirability bias or underreporting. This may result in over- or underreporting depending on the social norm. The CTC Youth survey, therefore, included both an honesty question and a fictitious drug to mitigate this bias by identifying inaccurate self-reports. Moreover, it should be noted that self-report data have been used extensively in similar research (Sturgis & Luff, 2021). Another potential limitation pertains to our use of multiple multi-level models. The substantial multicollinearity inherent among the capacity domains as well as a smaller community-level sample size precluded the feasibility of incorporating all domains simultaneously within a single model, despite the advantages such an approach could offer for elucidating their independent effects.

Despite these limitations, the study has several strengths, including a large sample size at the individual level and a comprehensive measure of community capacity that incorporates multiple dimensions. This detailed measurement allowed for a more nuanced understanding of the relationship between community capacity and substance use. Thus, the findings of this study provide important insights for the development of interventions and strategies aimed at reducing adolescent substance use. The identification of specific domains of community capacity that exhibit significant associations with substance use outcomes allows for strategic resource allocation and targeted capacity building efforts these areas. It is noteworthy that the statistically significant associations observed in this study are particularly remarkable considering that the capacity data and the outcome measures were obtained from separate data sources. It is unlikely that key informants were biased or influenced in their responses to the capacity items by knowledge of the outcome data.

In addition, research on the relationship between community capacity and health outcomes, particularly in the German context, is currently limited. While one case study analyzed capacity building within a health promotion intervention (Nickel et al., 2018) and one study assessed the effects of capacity on physical activity qualitatively (Loss et al., 2020), no quantitative study has examined the relationship between community capacity and health outcomes using a large sample in Germany. In addition, research on the association between community capacity and substance use among adolescents has been scarce, with only a few studies conducted in this area (Brown et al., 2014; Flewelling & Hanley, 2016). As such, the present study represents a novel and important contribution to this field, providing valuable new insights into the potential of community capacity to promote positive health outcomes among youth.

In particular, our results are consistent with the work of Flewelling and Hanley (2016), who found that higher levels of internal organization, structure, and community connections were associated with decreased alcohol consumption among adolescents. Our study corroborates this finding by demonstrating that prevention collaboration and sectoral-collaboration were linked to most substance use outcomes, emphasizing the importance of prevention coalitions connecting to other sectors within the community. Additionally, the results of this study align with findings on the effectiveness of capacity-building interventions such as the Communities That Care (CTC) program (Hawkins & Catalano, 1992), which have been shown to reduce substance use in adolescents.

## 6 Conclusion

In conclusion, the study found that higher community capacity is associated with lower odds of substance use, particularly for alcohol and tobacco use among students ranging from grades 5 to 11. These results suggest a link between higher levels of community capacity for prevention and reduced substance use among youth, implying the potential advantages of strengthening existing capacity building interventions, such as Communities That Care (CTC). Further longitudinal research is required to comprehend the underlying mechanisms through which community capacity is correlated to substance-use outcomes.

## Author contributions

**Vera Birgel:** Conceptualization, Methodology, Software, Formal analysis, Investigation, Data Curation, Writing - Writing - Original Draft, Review & Editing, Visualization; **Dominik Röding:** Conceptualization, Methodology, Formal analysis, Investigation, Writing - Review & Editing; **Maren Reder:** Conceptualization, Methodology, Software, Writing - Review & Editing; **Renate Soellner:** Conceptualization, Methodology, Software, Writing - Review & Editing, Supervision, Project administration; **Ulla Walter:** Conceptualization, Methodology, Writing - Review & Editing, Supervision, Project administration.

## Consent for publication

Not applicable.

## Availability of data and materials

The datasets generated and/or analyzed during the current study are not publicly available as they contain information that could compromise research participant privacy.

## Ethical approval and consent to participate

This study was conducted in accordance with the ethical principles outlined in the Declaration of Helsinki and has been approved by the Hannover Medical School's (9739_BO_K_2021) and University of Hildesheim's ethics committee. All participating communities completed an informed consenting process with a member of the research team. Written consent was obtained from the parents and students who agreed to participate in the Youth Survey. The consent form explicitly stated that participation was voluntary, and participants could withdraw from the study at any time without penalty or loss of benefits.

## Funding

The Federal Ministry of Education and Research (BMBF) supported the current project (funding number: 01EL2006A). The funding agency was not involved in the study design, manuscript writing, data collection or in the decision to submit it for publication.

## Trial registration

This study was registered with German Clinical Trial Register: DRKS00022819 on Aug 18, 2021.

## Declaration of competing interest

The authors declare that they have no known competing financial interests or personal relationships that could have appeared to influence the work reported in this paper.

## Data Availability

The data that has been used is confidential.
