# Supplementary material for: Contextual effects of community capacity as a predictor for adolescent alcohol, tobacco, and illicit drug use: A multi-level analysis
Source: SSM Popul Health. 2023 Sep 25;24:101521. doi: 10.1016/j.ssmph.2023.101521 (PMC10543175; doi:10.1016/j.ssmph.2023.101521)
Supplement: Multimedia component 1 [file mmc1.docx]

**Supplementary Material.**

**Supplementary Table 1**

Capacity measures and measurement scales.

| Capacity domain | Label | Survey item | Scale | Reliability | Item-test correlations |
| --- | --- | --- | --- | --- | --- |
| Total capacity score | Mean of capacity subscales | Prevention Collaboration  Sectoral-Collaboration  Knowledge & skills  Resources  Leadership  Cohesion  Problem-solving skills  Diversity  Needs orientation  Commitment | 1 = low;  4 = high | α=.78 | .21-.66 |
| Prevention collaboration | Sharing of information about prevention issues  Coordination of prevention issues  Agencies and organizations work together  Joint planning and decision-making    Network of people concerned about prevention issues  Clearly defined roles      Sharing of money and personnel | Organizations in (COMMUNITY) share information with each other about prevention issues.  Organizations in (COMMUNITY) coordinate prevention strategies.  Community agencies and organizations work together to address [problems with prevention strategies.  Organizations in (COMMUNITY) participate in joint planning and decision-making about prevention issues.  There is a network of people concerned about prevention issues who stay in touch with each other  In (COMMUNITY) each organization has a clearly defined role in carrying out the community’s prevention plan.  Organizations in (COMMUNITY) share money or personnel when addressing prevention issues | 1 = disagree;  4 = agree | α=.83 | .51-.71 |
| Sectoral-collaboration |  | In the past year, how much has [your organization] collaborated with [each sector] regarding prevention issues?   1. Secondary schools 2. Youth work 3. Youth centers 4. Social work services 5. Drug counseling centers 6. Police, judiciary 7. Child protection 8. Youth recreation 9. Juvenile justice 10. Human service agencies | 1 = not at all;  4 = a lot | α=.87 | .45-.69 |
| Knowledge & skills | Problems overwhelm local groups  Community is not accomplishing anything  What happens is matter of chance  People are knowledgeable about local prevention | Community problems overwhelm local community groups  My community never seems to be able to accomplish much at all.  What happens in (COMMUNITY) is largely a matter of chance  Generally, people in (COMMUNITY) are knowledgeable about local prevention efforts | 1 = disagree;  4 = agree | α=.65 | .32-.51 |
| Resources | Lack of financial resources      Lack of human resources      Lack of support      Financial conditions of the community | How much did a lack of financial resources pose barriers to prevention-related activities in your community over the past year?  How much did a lack of human resources pose barriers to prevention-related activities in your community over the past year?  How much did a lack of support in community pose barriers to prevention-related activities in your community over the past year?  How much of a problem are the current financial conditions of the community of [COMMUNITY]? | 1 = not at all;  4 = a lot | α=.69 | .31-.64 |
| Leadership | Lack of leadership | How much did a lack of leadership pose barriers to prevention-related activities in your community over the past year? | 1 = not at all;  4 = a lot | NA | NA |
| Cohesion | Tight-knit community  People care about community    Feeling of belonging | This is a tight-knit community?  Most people in (COMMUNITY) care a lot about the community  Most people in (COMMUNITY) tend to think of it as their home, the place they belong, rather than just a place to live. | 1 = not at all;  4 = a lot | α=.72 | .38-.54 |
| Problem-solving skills | Disagreements prevent addressing problems  Community has been successful    Respectful and satisfactory resolution of conflicts    Willingness to try new ideas for problem-solving | Disagreements in (COMMUNITY) prevent problems from being addressed  In the last year, my community has been successful at addressing social problems.  Community conflicts are usually resolved in a manner that is respectful of and satisfactory for the people involved.  This community is willing to try new ideas to solve community problems. | 1 = disagree;  4 = agree | α=.62 | .31-.35 |
| Inclusiveness | Participation of individuals from diverse ethnic and cultural backgrounds | In your community, how much do individuals from diverse ethnic and cultural backgrounds participate in prevention planning and implementation? | 1 = disagree;  4 = agree | NA | .35 |
| Needs orientation | Needs based prevention activities | Did your community select new prevention activities to respond to the community’s needs? | 1 = no,  2 = yes | NA | NA |
| Commitment | Commitment    Perceived efficacy in substance abuse prevention | Most people in (COMMUNITY) are committed to addressing community issues.  Most people in (COMMUNITY) think there is nothing they can do to prevent drug, alcohol, and tobacco abuse. | 1 = disagree;  4 = agree | r=.78 | .34 |

α = Cronbach‘s Alpha, r: Pearson correlation coefficient

**Supplementary Table 2**

Rotated component matrix of an EFA.

| Item description | Components | | | | | | | | | |
| --- | --- | --- | --- | --- | --- | --- | --- | --- | --- | --- |
|  | 1 | 2 | 3 | 4 | 5 | 6 | 7 | 8 | 9 | 10 |
| Coordination of prevention issues | **0.831** |  |  |  |  |  |  |  |  |  |
| Agencies and organizations work together | **0.808** |  | 0.311 |  |  |  |  | 0.432 |  |  |
| Sharing of information about prevention | **0.774** |  |  |  |  |  |  |  |  |  |
| Clearly defined roles | **0.660** |  |  |  |  |  |  |  |  |  |
| Network of people concerned about prevention | **0.634** |  |  |  |  |  |  | 0.382 |  |  |
| Joint planning and decision-making | **0.589** |  |  |  |  |  |  |  |  |  |
| Sharing of money and personnel | **0.558** |  | 0.338 |  |  |  | 0.474 |  |  |  |
| Youth work |  | **0.860** |  |  |  |  |  |  |  |  |
| Youth centers |  | **0.858** | 0.358 |  |  |  |  |  |  |  |
| Social work services |  | **0.830** |  |  |  |  |  |  |  |  |
| Drug counseling centers |  | **0.756** | 0.472 |  |  |  |  |  |  |  |
| Secondary schools |  | **0.750** | 0.387 |  |  |  |  |  |  |  |
| Human service agencies |  | **0.662** |  |  |  | 0.339 |  | 0.417 |  | 0.311 |
| Police, judiciary |  | **0.657** |  |  |  |  |  |  |  |  |
| Child protection |  | **0.652** |  |  |  |  |  | 0.385 |  |  |
| Juvenile justice |  | **0.580** |  |  |  |  |  |  | -0.340 | 0.372 |
| Youth recreation |  | **0.505** | -0.400 |  |  |  |  |  |  |  |
| What happens is matter of chance |  |  | **0.755** |  |  |  |  |  |  | -0.475 |
| Community is not accomplishing anything |  |  | **0.533** | -0.353 |  |  | 0.326 |  |  |  |
| People are knowledgeable about local prevention | 0.383 |  | **0.446** |  |  |  |  |  |  |  |
| Problems overwhelm local groups |  |  | **0.426** | -0.335 |  |  | 0.380 |  | 0.347 |  |
| Lack of human resources |  |  |  | **0.779** | 0.327 |  |  |  |  |  |
| Lack of support |  |  |  | **0.734** |  |  |  |  |  |  |
| Financial conditions of the community |  |  |  | **0.732** |  |  |  |  |  |  |
| Lack of financial resources |  |  |  | **0.625** | 0.499 |  |  |  |  |  |
| Lack of leadership | -0.390 |  |  |  | **0.634** |  |  |  |  |  |
| Feeling of belonging |  |  |  |  |  | **0.839** |  |  |  |  |
| People care about community |  |  |  |  |  | **0.806** |  |  |  |  |
| Tight-knit community |  |  |  |  | -0.352 | **0.564** |  |  |  | 0.332 |
| Disagreements prevent addressing problems |  |  |  |  |  |  | **0.808** |  |  |  |
| Willingness to try new ideas |  |  |  |  |  |  | **0.738** |  | -0.397 | 0.418 |
| Respectful and satisfactory resolution of conflicts |  |  |  |  |  |  | **0.578** |  |  |  |
| Community has been successful |  |  | 0.370 |  |  | 0.333 | **0.470** |  |  |  |
| Participation from diverse backgrounds | 0.312 |  |  | 0.356 | -0.327 |  |  | **0.682** | -0.374 |  |
| Needs based prevention activities |  |  |  | 0.377 | -0.392 |  | -0.420 |  | **0.687** |  |
| Commitment |  |  | 0.385 | 0.365 | 0.304 |  |  |  |  | **0.566** |
| Perceived efficacy |  |  |  |  |  | -0.348 |  | -0.315 |  | **0.498** |

Data in bold indicate p < .001

Factor loadings below 0.3 are masked out.

**Supplementary Table 3**

Results of the confirmatory factor analysis of the community capacity subscales.

| **Scale Items** | **Factor Loading** | **p** |
| --- | --- | --- |
| *Prevention collaboration*   1. Sharing of information about prevention 2. Coordination of prevention issues 3. Agencies and organizations work together 4. Joint planning and decision-making 5. Network of people concerned about prevention 6. Clearly defined rolls 7. Sharing of money and personnel | 0.821  0.767  0.678  0.645  0.590  0.528  0.532 | < .001  < .001  < .001  < .001  < .001  < .001  < .001 |
| *Sectoral-Collaboration*   1. Secondary schools 2. Youth work 3. Youth centers 4. Social work services 5. Drug counseling centers 6. Police, judiciary 7. Child protection 8. Youth recreation 9. Juvenile justice 10. Human service agencies | 0.724  0.728  0.531  0.746  0.699  0.600  0.551  0.498  0.495  0.408 | < .001  < .001  < .001  < .001  < .001  < .001  < .001  < .001  < .001  < .001 |
| *Knowledge & Skills*   1. Problems overwhelm local groups 2. Community is not accomplishing anything 3. What happens is matter of chance 4. People are knowledgeable about local prevention | 0.747  0.569  0.474  0.459 | < .001  < .001  < .001  < .001 |
| *Resources*   1. Lack of financial resources 2. Lack of human resources 3. Lack of support 4. Financial conditions of the community | 0.714  0.638  0.599  0.501 | < .001  < .001  < .001  < .001 |
| *Cohesion*   1. Feeling of belonging 2. People care about community 3. Care about community | 0.886  0.599  0.656 | < .001  < .001  < .001 |
| *Problem-solving skills*   1. Disagreements prevent addressing problems 2. Perceived efficacy 3. Respectful and satisfactory resolution of conflicts 4. Willingness to try new ideas | 0.702  0.537  0.499  0.555 | < .001  < .001  < .001  < .001 |

**Supplementary Table 4**

Results of the correlation analysis on the community level (n=28).

|  | (1) | (2) | (3) | (4) | (5) | (6) | (7) | (8) | (9) | (10) | (11) |
| --- | --- | --- | --- | --- | --- | --- | --- | --- | --- | --- | --- |
| (1) Total capacity score |  | r=.66  p<.001 | r=.21  p=.042 | r=.79  p<.001 | r=.28  p<.001 | r=.24  p<.001 | r=.49  p<.001 | r=.58  p<.001 | r=.28  p<.001 | r=.29  p<.001 | r=.52  p<.001 |
| (2) Prevention collaboration |  |  | r=.23  p<.001 | r=46  p<.001 | r=12  p<.001 | r=16  p<.001 | r=.14  p<.001 | r=.40  p<.001 | r=.73  p<.001 | r=.20  p<.001 | r=.09  p<.001 |
| (3) Sectoral collaboration |  |  |  | r=.03  p=001 | r=.18  p<.001 | r=.04  p<.001 | r=.60  p<.001 | r=.22  p<.001 | r=.15  p<.001 | r=.14  p<.001 | r=-.58  p<.001 |
| (4) Knowledge |  |  |  |  | r=.28  p<.001 | r=.38  p<.001 | r=.20  p<.001 | r=.64  p<.001 | r=.02  p=.114 | r=-.17  p<.001 | r=.38  p<.001 |
| (5) Resources |  |  |  |  |  | r=.74  p<.001 | r=.24  p<.001 | r=.19  p<.001 | r=.25  p<.001 | r=.56  p<.001 | r=.13  p<.001 |
| (6) Leadership |  |  |  |  |  |  | r=.14  p<.001 | r=.30  p<.001 | r=.08  p<.001 | r=.11  p<.001 | r=.19  p<.001 |
| (7) Cohesion |  |  |  |  |  |  |  | r=.02  p=.15 | r=.15  p<.001 | r=-.20  p<.001 | r=.78  p<.001 |
| (8) Problem-solving skills |  |  |  |  |  |  |  |  | r=.05  p<.001 | r=-.16  p<.001 | r=.04  p<.001 |
| (9) Inclusiveness |  |  |  |  |  |  |  |  |  | r=.19  p<.001 | r=.02  p=.991 |
| (10) Needs orientation |  |  |  |  |  |  |  |  |  |  | r=-.38  p<.001 |
| (11) Commitment |  |  |  |  |  |  |  |  |  |  |  |
